# Stem cell activation in skeletal muscle regeneration

**DOI:** 10.1007/s00018-014-1819-5

**Published:** 2015-01-09

**Authors:** Xin Fu, Huating Wang, Ping Hu

**Affiliations:** 1 State Key Laboratory of Cell Biology, Institute of Biochemistry and Cell Biology, Shanghai Institutes for Biological Sciences, Chinese Academy of Sciences, 320 Yueyang Road, Shanghai, 200031 China; 2 Department of Obstetrics and Gynaecology, Li Ka Shing Institute of Health Sciences, Prince of Wales Hospital, The Chinese University of Hong Kong, Hong Kong SAR, China

**Keywords:** Satellite cells, Quiescent stem cells, Epigenetics, Non-coding RNA, DNA methylation, Growth factors

## Abstract

Muscle stem cell (satellite cell) activation post muscle injury is a transient and critical step in muscle regeneration. It is regulated by physiological cues, signaling molecules, and epigenetic regulatory factors. The mechanisms that coherently turn on the complex activation process shortly after trauma are just beginning to be illuminated. In this review, we will discuss the current knowledge of satellite cell activation regulation.

## Introduction

Skeletal muscle is voluntarily controlled striated muscle tissue that produces locomotion, postural behavior, and breathing. It is also the largest insulin-stimulated glucose utilization tissue in the body [1]. As the most abundant tissue in the human body, on average, it accounts for 40–50 % of an adult male’s and 30–40 % of an adult female’s body weight. Maintenance of muscle mass is not only critical for precise movements, but also important for optimal metabolic homeostasis. Unfortunately, due to the function and location of skeletal muscle, it is susceptible to the damage caused by overstretching, straining, trauma, everyday wear and tear, and several degenerative muscle disorders. These damages can be repaired through muscle regeneration mediated by muscle stem cells. Satellite cells represent a major group of muscle stem cells. Initially identified by Mauro [2] in 1961, satellite cells are located between the sarcolemma and the basal lamina of myofibers. These cells usually remain quiescent with a large nuclear-to-cytoplasmic ratio and a low number of mitochondria [3]. In response to exercise and injury, quiescent satellite cells are activated to enter the cell cycle, proliferate, and eventually exit at G_1_, fusing to form terminally differentiated multinucleated myofibers.

In addition to satellite cells, several other types of muscle-resident adult stem cells have recently been found [3]. These stem cells are also capable of muscle lineage differentiation and their activation also represents an important part of muscle regeneration, although the regulatory mechanism remains largely unknown.

There are many sophisticated reviews on satellite cells and muscle regeneration [3–12]. Here, we summarize the current literature on regulation of satellite cells and other muscle-resident stem cell activation.

## Satellite cells and satellite cell activation

Although multiple types of stem cells with muscle lineage differentiation potential have been identified [13], satellite cells are the major contributor to the remarkable regenerative capabilities of skeletal muscle. Satellite cells were initially discovered by Alex Mauro more than 50 years ago using electron microscopy, as mononucleated cells located at the periphery of muscle fibers [2]. Mauro suggested that satellite cells “might be pertinent to the vexing problem of skeletal muscle regeneration” [2]. Indeed, later experiments revealed that satellite cells were able to give rise to terminally differentiated multinucleated myotubes through cell fusion to regenerate damaged myofibers [14–20].

During embryonic development, satellite cells emerge together with the muscle in which they reside and share the same origin as muscle. Satellite cells from the trunk and limb muscles originate from the dermomyotome, while the majority of the satellite cells of the craniofacial muscles are derived from the head mesoderm [4, 21–24]. The number of satellite cells reaches a peak at the neonatal stage, accounting for about 30–35 % of the total myofiber nuclei. The number decreases to ~2–7 % of the total myofiber nuclei in adulthood [25–29].

Satellite cells are activated and are more proliferative during the neonatal period to support the rapid gain in muscle mass [25, 30–32]. In contrast to the situation in neonates, the majority of satellite cells are mitotically quiescent in adults, remaining at the G_0_ stage, although the mechanism by which active satellite cells become quiescent after the burst of postnatal muscle mass growth is not clear yet.

**Fig. 1:**
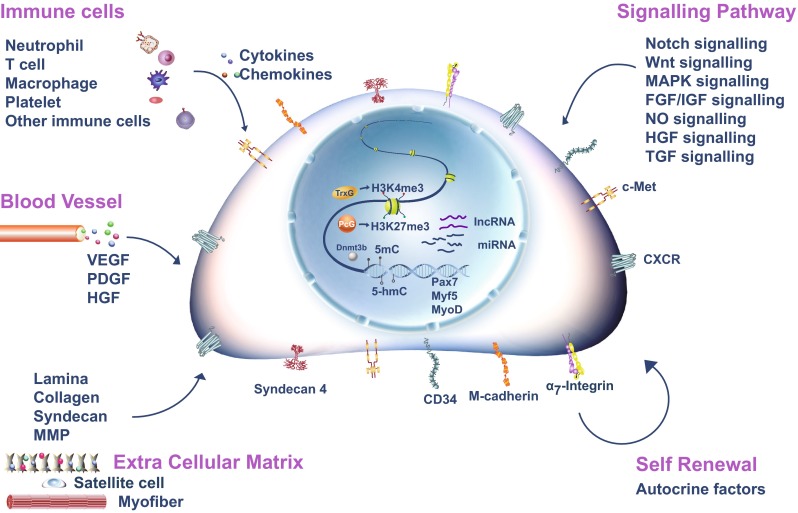
Regulation of satellite cell activation. Satellite cell specific surface markers were indicated on the cell membrane. Key transcription factors involved in satellite cell activation were indicated in the nuclei of satellite cell. Key signaling pathways regulating satellite cell activation were listed. Major microenvironment components including extra cellular matrix and neighboring cell types (immune cells and blood vessels) affecting satellite cell activation were illustrated

The quiescent satellite cells reside in a unique niche in intact muscles [5]. They are located closely juxtaposed between the sarcolemma of muscle fibers and the basal lamina that surrounds the fiber [2]. These cells display specific gene expression profiles compared to actively proliferating satellite cells. Pax7, Pax3, M-cadherin, Syndecan-4, CD34, α_7_-Integrin, and CXCR4 [33–35] dominantly express in quiescent satellite cells (Fig. 1), and MyoD expression is absent in quiescent satellite cells [36, 37]. They can be quickly activated to re-enter the cell cycle and proliferate in response to extrinsic signals, a process referred to as satellite cell activation.

The morphology of activated satellite cells is different from that of quiescent satellite cells. Quiescent satellite cells are usually spindle-shaped with little cytoplasm and few organelles [38, 39], whereas activated satellite cells are larger with an expanded cytoplasm and more organelles [39, 40]. The earliest marker for activated satellite cells is phosphorylated p38, followed by MyoD [41–43]. Although detected in the majority of quiescent satellite cells, Myf5 is highly upregulated on activation [44, 45]. During G1 phase, shortly after exit from quiescence, MyoD does not promote differentiation, but instead directly regulates the expression of Cdc6, a gene involved in rendering chromatin accessible for DNA replication, allowing cell cycle entry [43, 46].

MyoD plays multiple roles at different stages of muscle differentiation in a context-dependent manner. For example, MyoD expressed in quiescent cells has been reported to inhibit cell proliferation in a differentiation-independent way [47]. In myoblasts, MyoD can also inhibit cell cycle entry by the induction of p21 and upregulation of apoptotic genes [48, 49]. After the first cell division, the proliferative cells enter the cell cycle and continue to divide every 10 h [50].

Upon stimulation, such as muscle damage, exercise, or pathogenic conditions, the satellite cells start to proliferate and give rise to a myogenic precursor cell population called myoblasts. Myoblasts can go through several rounds of amplification, then exit the cell cycle and fuse to each other or to the existing myofibers to form terminally differentiated myofibers. Some of the progeny of the activated satellite cells can restore the pool of quiescent stem cells by asymmetric self-renewal [51]. ERK signaling regulates the reversible quiescence of a subpopulation of satellite cells through ERK signaling inhibitor Spry1 [52]. Depletion of Spry1 in satellite cells increases the number of cells committed to apoptosis and reduced the number of quiescent satellite cells after muscle injury reparation, suggesting that Spy1 is required for reversible quiescence [52]. Activation of satellite cells is the critical step in the initiation of muscle regeneration. It is subjected to multiple layers of tight regulation. Physiological cues, signaling molecules, and epigenetic regulators are all involved in the orchestration of the orderly activation of satellite cells upon stimulation.

## Physiological cues to induce satellite cell activation

### Disruption of muscle fibers and basal lamina

The intact myofiber sarcolemma and the basal lamina provide an important niche to maintain the quiescent state of satellite cells [11]. They are destroyed in muscle injury, leading to disruption of the protective niche for quiescent satellite cells. Damages to the basal lamina destroy the collagen–laminin network, where satellite cells anchor themselves through α7/β1 integrins [53]. The mobilization of satellite cells contributes to their activation. Furthermore, destruction of the sarcolemma and the basal lamina allows an influx of calcium and the release of hepatocyte growth factor (HGF) from the extra cellular matrix (ECM) to directly activate the unprotected satellite cells [54–58].

Muscle damage also generates a large amount of destructed muscle fibers. Selective induction of fiber damage using bupivacaine, without disruption of the basal lamina and other cell types, results in elevated satellite cell activation and proliferation [59], suggesting that the damaged fibers provide signals that activate quiescent satellite cells. Indeed, these dying fibers produce nitric oxide (NO) that further activates HGF and downstream signaling to induce satellite cell activation [58]. Therefore, both disruption of the protective niche and the factors released by dying fibers contribute to the activation of quiescent satellite cells.

### Infiltration of immune cells

Muscle injury, stretching, overuse, and degenerative muscle diseases induce the infiltration of large amounts of immune cells, initiated by early neutrophil invasion and followed by macrophage infiltration [60–62]. Chemokines, cytokines, and growth factors produced by macrophages, together with fibroblasts, attract more circulating inflammatory cells, including T cells and B cells. Cytokines, growth factors, and chemokines secreted by these inflammatory cells, such as IL-6 and IFNγ, can promote satellite cell activation and proliferation [50, 63–65]. Inhibition of inflammation by non-steroidal anti-inflammatory drugs in humans reduces the number of activated satellite cells, thus slowing down muscle regeneration [66–68]. Immune cells and the inflammation reaction followed by immune cell infiltration provide a microenvironment for satellite cell activation and proliferation (Fig. 1).

### Blood vessels

Upon muscle injury, blood vessels are also severely disrupted. The vascular endothelial cells of damaged vessels release growth factors such as vascular endothelial growth factor (VEGF), platelet-derived growth factor (PDGF), and HGF [13]. These factors promote satellite cell activation and proliferation [11]. Vascularization and angiogenesis are essential steps in muscle regeneration. Many growth factors enriched at the injury site to promote vessel restoration can also promote satellite cell proliferation [69, 70]. The restored blood vessels then signal the activated satellite cells to return to the quiescent state [71]. Communication between satellite cells and blood vessels can, thus, regulate the dynamic cycle of satellite cell activation and quiescence maintenance.

### Signals to activate satellite cells

Upon muscle injury, a combination of signals is generated by damaged myofibers, blood vessels, and immune cells to wake up the quiescent satellite cells. The activated satellite cells also signal back to the environment to orchestrate orderly muscle regeneration (Fig. 1).

### HGF

HGF is a mesenchyme-derived heparin-binding glycoprotein that regulates cell proliferation, cell survival, cell motility, and morphogenesis [72]. HGF can bind to the c-Met receptor to regulate cell growth, cell motility, morphogenesis, and organ regeneration by activating a tyrosine kinase signaling cascade [73]. Mice deficient in HGF or its receptor, c-Met, lack all the muscle groups derived from migratory hypaxial precursor cells [57, 74]. The application of exogenous HGF to somites induces ectopic delamination of myogenic precursor cells into the lateral plate mesoderm [75, 76]. These results suggest that HGF is essential in inducing the migration of myogenic precursor cells in embryonic myogenesis.

Satellite cells express both HGF and c-Met. Muscle cells and non-muscle cells in close proximity produce and secret HGF, which is sequestered in the ECM around intact muscle fibers [39, 54, 58]. Upon muscle damage, HGF is released from the ECM, promoting the entry of quiescent satellite cells into cell cycle [54–56]. Satellite cells also express HGF; therefore, the activation of satellite cells by HGF can undergo both paracrine and autocrine [56].

The presence of HGF-bound c-Met has been considered to be the initial step in satellite cell activation [40, 55]. Recently, Rodgers et al. showed that injury-related systemic signals could induce the quiescent satellite cells to transit from G_0_ to G_Alert_. The G_Alert_ cells are primed for activation. HGF, c-Met, and the downstream mTORC1 signaling are required for this transition [77]. The addition of HGF to satellite cells cultured with single fibers induces prominent satellite cell activation [40, 56, 78], further indicating the pivotal role of HGF in this process. HGF could respond to weak signals induced by injury and prime the quiescent satellite cells for the “alert” stage. If the injury continues and the induced systemic signals cross the threshold, the alerted satellite cells will be quickly activated and muscle injury will be efficiently repaired.

The molecular mechanism by which HGF further activates satellite cells has not been fully elucidated. It may activate the downstream tyrosine kinase signaling pathway to alter the expression levels of cell cycle-related genes. The expression levels of *c*-*fos* and *c*-*jun*, the early genes of tyrosine kinase signaling, are rapidly upregulated in satellite cells 3–6 h after muscle injury. This is considered to be the immediate response to HGF-c-Met-mediated signaling. Receptor-bound HGF can also increase Twist expression [79] which further activates EMT (epithelial mesenchymal transition) [80]. Meanwhile HGF-mediated downregulation of the protein levels of p27kip1 in a p21Cip1/Waf1-independent manner in satellite cells [79], helps the cells to overcome cell cycle blockage. HGF can activate satellite cells rapidly after trauma by promoting EMT to release them from their quiescent prone niche and removing cell cycle blockages.

### NO

NO is a freely diffusible small messenger capable of pleiotropic cellular functions, such as survival, stress resistance, and neurotransmission [81]. NO is produced in skeletal muscle through reactions catalyzed by nitric oxide synthase (NOS). Within 6 h post-injury, NOS mRNA levels are significantly increased in both damaged muscle fibers and the infiltrating macrophages, therefore elevating the NO levels at the injury site [82–84]. In iNOS^(−/−)^ mice, satellite cells fail to proliferate and differentiate after injury [85], suggesting that NO is required for normal muscle reparation after injury.

NO plays multiple roles during the muscle regeneration process. At the early stage of muscle damage, it promotes macrophages to lyse damaged muscle cells in a reactive oxygen species (ROS)-independent manner to protect cells from further ROS damage [86], and stimulates the release of HGF, together with other growth factors and cytokines to activate satellite cells [87]. At the second stage of muscle regeneration, NO inhibits neutrophil-mediated lysis of muscle cells and reduces ROS generated from prolonged inflammation, protecting the activated satellite cells from ROS stress and apoptosis [82].

NO activates satellite cells not only by facilitating the release of HGF, but also by antagonizing the inhibitory effects of TGF-β on satellite cells. The administration of L-NAME, an NOS inhibitor, at the injury site in rat muscle leads to abnormally elevated TGF-β level that induces fibrosis [88].

### IGF and FGFs

Insulin-like growth factor (IGF) is a circulating hormone critical for development and regeneration of almost every organ [89]. IGF signaling is initiated by binding of IGF to the IGF receptor (IGFR) to activate its tyrosine kinase activity and autophosphorylation, which in turn phosphorylates insulin receptor substrate 1 (IRS-1). Phosphorylated IRS-1 recruits the regulatory subunit of PI3K and activates it. Activated PI3K phosphorylates Akt, which then activates mTOR and p70S6 kinase to turn on the IGF-PI3K/Akt-mTOR-S6K axis of signaling pathway. This signaling process has been shown to be important for muscle mass maintenance [90]. Six IGF binding proteins, named IGFBP1-6, bind IGF in the extracellular fluid and the circulation to further regulate IGF activities [91]. The expression of IGF and all six IGFBPs has been detected in regenerating skeletal muscle [92], suggesting their roles in muscle wound healing.

Muscle damage induces the expression of alternative splicing isoforms of IGF named mechano-growth factor (MGF) and IGF-IEa [93]. MGF is only expressed in the damaged muscle and its expression is correlated with the activation of quiescent satellite cells [94]. IGF-IEa is expressed later than MGF during muscle regeneration, correlating with myoblast proliferation and differentiation [95, 96]. MGF elevates the activity of superoxide dismutase, the enzyme required for decreasing the level of ROS [97], thus protecting the satellite cells from ROS-induced damage. IGFBP6 is an IGF sequester, which increases the expression levels of IGF isoforms. However, its expression level is dramatically decreased at the early stage of muscle regeneration to allow more IGF available to activate satellite cells and promote their proliferation [98]. IGF-IR heterozygous mice display decreased the levels of MyoD expression and satellite cell activation [99], further confirming the importance of IGF in the satellite cell activation process.

The mechanism of IGF-mediated satellite cell activation has not been fully elucidated but may involve the upregulation of Myf5 expression upon injury. After muscle injury, an influx of calcium triggers calcineurin and calmodulin kinase through calcium binding to calmodulin, to activate Myf5 expression. IGF can activate Myf5 through the calcium-mediated activation pathway [100]. In addition, it could also activate Myf5 expression through PI3K/Akt and ERK signaling pathways [100, 101]. It can also activate expression of cyclin D2 to promote entry to cell cycle and cell proliferation through MEK/ERK and PI3K/AKT signaling pathways in C2C12 cells. IGF2 expression levels could be regulated by N-cadherin signaling through activation of p38α and β [102]. An immunoglobulin superfamily member C_do_ can cooperate with the scaffold protein JLP to increase the level of active p38α and β, thus increasing the expression level of IGF2 during the myoblast differentiation process [103]. The similar mechanism may also contribute to the activation of satellite cells by IGF. Another immunoglobulin superfamily member, the receptor for advanced glycation end-products (RAGE) activated by HMGB1, also plays an important role in satellite cell activation. RAGE is transiently expressed in satellite cells located in injured muscles and represses Pax7 expression through activation of p38-MAPK signaling [104–107]. Deletion of RAGE in muscles leads to increased satellite cell number and Pax7 expression level [106], suggesting that RAGE is required for proper timing of muscle regeneration. RAGE may also be able to regulate satellite cell activation by repressing Pax7 expression.

Fibroblast growth factors (FGFs) belong to the family of heparin-binding circulating mitogens that regulate cell survival, proliferation, migration, differentiation, and morphogenesis. The expression of many FGF family members has been detected in skeletal muscle [108]. Among them, FGF6 has been shown to be expressed predominantly in myogenic cells, and its expression level is upregulated during muscle regeneration. In FGF6^−/−^ mice, the number of activated satellite cells is significantly reduced and the size of quiescent satellite cells pool remains constant, suggesting that FGF6 is essential for satellite cell activation and proliferation [109]. Consistent with the results from gene knockout experiments, the addition of exogenous FGF1, 2, 4, 6, or 9 stimulates satellite cell proliferation significantly in vitro. The stimulating effects of FGF2, 4, 6, and 9 are further enhanced in combination with HGF [110, 111].

FGFs activate the downstream signaling pathways through binding to FGF receptor 1–4 (FGFR1-4), which are transmembrane tyrosine receptors. All four FGFRs are detected in satellite cells. Among them, FGFR1 and 4 are the most prominent ones [110]. The expression level of FGFR1 is significantly upregulated shortly after muscle injury, correlating with the activation of satellite cells [110]. Overexpression of FGFR1 facilitates the proliferation of cultured myoblasts and represses myoblast differentiation [112]. It may also be able to promote the proliferation of activated satellite cells.

The binding of FGF to FGFR leads to dimerization and autophosphorylation of the receptor, followed by activation of Ras signaling pathways. Overexpression of constitutively activated Ras can bypass FGF, promoting myoblast proliferation [113]. FGF can also activate MKK-ERK signaling cascade, facilitating the transition from G_1_ to S phase in myoblasts, thus increasing their proliferation [114]. FGF can also induce the activation of satellite cells by enhancing the G_1_-S transition through ERK signaling pathway.

Similar to IGFs, FGF can also regulate calcium-mediated signaling. IGF enhances intracellular calcium intake in CD34^+^ satellite cells, as indicated by lighting up cells with the calcium-sensitive fluorescent dye X-Rhod-1. The elevated intracellular calcium level induced by FGF2 triggers nuclear translocation of NFATc3 and NFATc2, which facilitates MyoD expression in satellite cells leading to their activation. The effects of IGF on the calcium intake of satellite cells and satellite cell activation are antagonized by the blockage of the TRPC ion channel [115].

### Notch

Notch signaling is one of the major regulatory pathways in cell fate determination. Notch is a family of transmembrane receptors containing four members, Notch1–4. After binding to its ligands (Delta-like 1, Delta-like 4, Jagged 1, and Jagged 2), Notch undergoes protease cleavage to free its intracellular domain, NICD, a transcription coactivator that facilitates RBP-J kappa (Rbpj, the Notch target transcription factor) -mediated transcription activation [116, 117]. In embryonic muscle development, Notch signaling is required for myogenic cell fate commitment and muscle stem cell maintenance. Notch ligand Delta 1 (Dll1) null mutant mice displayed hypotrophy due to premature differentiation of satellite cells [118]. Consistent with it, muscle-specific depletion of Rbpj leads to loss of myogenic stem cells due to increasing differentiation during embryogenesis [119]. Muscle-specific overexpression of NICD increases Pax7^+^ muscle stem cell numbers and maintains these cells in an undifferentiated state [120].

Notch1, 2, 3, Notch/Rbpj targets Hey1, HeyL, and Hes1 are all expressed robustly in quiescent satellite cells. Disruption of Notch signaling by Rbpj depletion in satellite cells leads to precocious differentiation and depletion of satellite cells [121]. All these genetic data support that Notch signaling is essential for maintenance of satellite cell quiescence, and it should be downregulated in satellite cell activation. Indeed, endogenous Notch signaling is dramatically reduced in activated satellite cells isolated from regenerating muscle, compared with quiescent satellite cells [121–123]. This reduction in activity occurs within 20 h of an injury [122]. The first mitosis of activated satellite cells after injury occurs at about the same time (18-24 h) [50]. The correlation of the timing of Notch signaling downregulation and the cell cycle entrance of quiescent satellite cells indicates that these two events may be tightly coupled.

Notch signaling has also been reported to be required for satellite cell proliferation. An abnormally high numbers of satellite cells are found in Notch3 knockout mice [124]. Activation of Notch signaling in cultured satellite cells also promotes proliferation of these cells [125, 126]. These seemingly contradictory results can be explained by the presence of two waves of Notch signaling during the activation of satellite cells. The first wave maintains the quiescent state of the satellite cells, which is inactivated upon cell activation. The second wave is turned on at the proliferative stage of the activated satellite cells. The changes in Notch signaling levels during muscle regeneration support this notion. Notch signaling level decreases almost immediately after muscle injury when satellite cells are activated [122], and increases again 4–5 days post-injury [127].

Most of the current observations about the function of Notch in satellite cell activation are correlations. Whether Notch is just a passive downstream responder of satellite cell activation or actively causes the activation is still under debating. To clarify this issue, continuous monitoring of Notch signaling level during the satellite cell activation process and additional well-controlled manipulations of Notch signaling at various stages of satellite cell activation are needed.

### Wnt

Wnt proteins are soluble signaling molecules regulating multiple cellular processes, including cell fate determination, stem cell proliferation, cell polarity, morphology, and tumorigenesis. The canonical Wnt signaling cascade is turned on by the binding of Wnt ligand to transmembrane receptors Frizzled and low-density lipoprotein receptor-related protein co-receptor (LRP). Ligand binding stimulates the phosphorylation of Dishevelled (Dsh) and inactivates GSK3β, triggering the stabilization of the common downstream Wnt effecter β-catenin. When Wnt signaling is off, a destruction complex composed of GSK3β, Adenomatous polyposis coli (APC), and Axin2 associates with β-catenin to drive its ubiquitination and degradation. When Wnt signaling is on, β-catenin accumulates due to the disassembly of the destruction complex and is translocated into nucleus to serve as a coactivator for TCF/LEF transcription factors to activate the expression of target genes [128].

Wnt signaling is upregulated upon muscle injury. There is increased TCF reporter activity in myogenic cells two days post-muscle injury [125]. Exogenous Wnt1, 3a, and 5a promote satellite cell proliferation, whereas Wnt4 and 6 inhibit it [3]. β-catenin can promote satellite cell self-renewal and prevent immediate satellite cell differentiation [125, 129, 130]. Consistent with the notion that Wnt promotes satellite cell proliferation, nuclear localization of β-catenin has been detected only in activated satellite cells and myoblasts, but not in differentiating muscle cells (myogenin^+^). Perplexingly, other observations seem to oppose the pro-proliferative functions of Wnt. When β-catenin expression level is reduced by RNAi in satellite cells, more activated Pax7^+^MyoD^+^ satellite cells are observed, whereas constitutive expression of β-catenin leads to downregulation of MyoD [130], suggesting that β-catenin inhibits satellite cell activation. In aged mice, elevated serum Wnt level inhibits the proliferation of satellite cells and directs their fate toward the fibrogenic lineage. Furthermore, injection of Wnt inhibitors Dkk1 and sFRP3 in aged mice leads to reduction of fibrogenic lineage differentiation [131, 132]. These seemingly contradictory results could be due to different systems used in the experiments and potentially altered Wnt signaling pathways in aged animals.

In addition to the canonical Wnt pathway, Wnt can also activate Rho/Rac and JNK signaling through crosstalk mediated via Dsh, to regulate cell polarity [133]. This non-canonical Wnt signaling functions in satellite cells. Wnt7a stimulates symmetrical division of satellite cells to expand the activated satellite cell pool via activation of planar cell polarity (PCP) signaling. The satellite cell number increases dramatically upon Wnt7a overexpression, whereas the depletion of Wnt7a leads to a marked decrease of satellite cell number [134]. The expansion of the activated satellite cell pool induces the expression of fibronectin, which further modifies the satellite cell niche and stimulates Wnt7a-Frizzled 7-PCP signaling to form a feedback loop during muscle regeneration [135].

Both canonical and non-canonical Wnt are involved in the regulation of satellite cell activation and proliferation. Experiments, that can pinpoint Wnt functions at a particular cell stage, i.e., the initial stage of satellite cell activation, activated satellite cell stage, myoblast stage etc., will help to elucidate the functions of Wnt signaling during satellite cell activation.

### TGF-β

The transforming growth factor beta (TGF-β) superfamily comprises many secreted factors essential in nearly every aspect of cellular behavior. It is grouped into subfamilies based on sequence homology, including the TGF-β, activin, glial cell line derived neurotrophic factor (GDNF), growth/differentiation factor (GDF), and bone morphogenetic protein (BMP) subfamilies. TGF-β ligands bind to type I and II serine/threonine kinase cell surface receptors. Upon ligand binding, the type II receptor phosphorylates the type I receptor to activate it. The activated type I receptor subsequently phosphorylates receptor-regulated Smads (R-Smads), which forms a heterodimer with common mediator Smad (co-Smad) to mediate its nuclear translocation and transcription activation [136].

In general, TGF-β signaling plays a negative role in the regulation of myogenesis by repressing the expression of MyoD and myogenin [137–140]. It is highly expressed in quiescent satellite cells and repressing cell cycle progression [98]. Myostatin is a TGF-β family member expressed specifically in muscle tissue to prevent muscle growth and differentiation [141]. Myostatin maintains satellite cell quiescence and represses satellite cell self-renewal by inducing p21^CIP^ expression [142]. Myostatin represses the expression of MyoD to prevent satellite cells from activation [143]. It can also increase Pax7 expression level through ERK signaling pathway to help maintaining satellite cell quiescence [144]. Many growth factors, including IGF and FGF, as described above, antagonize the inhibitory effects of TGF-β to activate satellite cells during muscle regeneration, although the mechanisms remain to be defined.

TGF-β level is elevated in circulation with aging, preventing satellite cells from entering into cell cycle by inducing the expression of cell cycle inhibitors such as p15, p16, p21, and p27 [145]. The injection of an antibody against TGF-β at the injury site of aged mice rejuvenates satellite cells by promoting their activation [145].

### Extracellular matrix signals

Satellite cells have close contact with ECM. In addition to intracellular responses to signaling molecules, remodeling of ECM has been shown to be a critical step in satellite cell activation. Quiescent satellite cells express ECM components such as versican, fibrillin-2, and glypicans. These ECM proteins bind HGF and other growth factors to lower their effective concentration around satellite cells. Once trauma occurs in a muscle, ECM is damaged and the trapped growth factors are released to activate the quiescent satellite cells (Fig. 1).

### Syndecans

Heparin sulfate proteoglycans (HSPGs) are composed of two or three linear polysaccharides covalently attached to various proteins at the cell surface and in ECM. They act as adhesion proteins and receptors for many growth factors, morphogens, and adhesion proteins [146]. Transmembrane syndecans are one of the major forms of membrane HSPGs. Satellite cells specifically express syndecan-3 and -4 [147], which serve as low affinity FGF receptors [148]. Satellite cell activation and proliferation is abolished in syndecan-4 knockout mice due to loss of MAPK signaling [149], showing syndecan-4 to be required for satellite cell activation. Syndecan-4 can also form a co-receptor complex with Frizzled-7 to facilitate Wnt7-mediated non-canonical Wnt signaling to regulate satellite cell activation [135]. In contrast, satellite cell activation is accelerated in syndecan-3 knockout mice, although the cells are arrested in S phase [149]. Syndecan-3 and -4 may work together to regulate the coherent chain of reactions during satellite cell activation and proliferation.

### MMPs

Matrix metalloproteinases (MMPs) belong to a family of zinc-dependent endopeptidases capable of degrading ECM proteins. In skeletal muscle, MMPs are released at the reparation site by damaged myofibers or immune cells recruited by muscle injury [150]. MMP2 is produced by satellite cells and damaged myofibers [151–153], while MMP9 is generated by infiltrating leukocytes and macrophages [153]. NO induces the increase of the enzymatic activity and expression levels of MMP2 and MMP9. Both MMP2 and 9 cleave collagens, laminin, and other ECM components to release HGF from the sequestered sites to activate satellite cells [154, 155].

Inhibitors of TIMPs (metalloproteinases) are also involved in satellite cell activation. TIMP3 is highly expressed in quiescent satellite cells and downregulated in activated satellite cells [98]. Consequently, the activity of MMP2 is elevated in activated satellite cells [151–153]. In addition to MMP2, TIMP3 can also repress the activity of TNF-α converting enzyme to block TNF-α mediated p38 signaling pathway activation and further inhibit satellite cell activation [156].

### Pax7 and Pax3 in satellite cell activation

Pax7 and Pax3 are satellite cell markers. Pax3 is expressed in satellite cells lying in most trunk and forelimb, but not hindlimb muscles [157], whereas Pax7 is expressed in satellite cells resident in all muscles [158]. The expression of Pax7, but not Pax3, prevents satellite cells from undergoing apoptosis [159]. Heterogeneity has been demonstrated in satellite cells. Some satellite cells express high levels of Pax7 (Pax7^High^), whereas ~3 % of satellite cells express low levels (Pax7^Low^) [98]. The Pax7^High^ cells take longer to enter the cell cycle after satellite cell activation [11]. Whether the Pax7^High^ and Pax7^Low^ satellite cells are activated in distinct patterns remains unclear.

Pax3 and Pax7 can control the expression of Myf5 and MyoD [160], but how Pax7/3 activates MyoD upon muscle injury, in particular, the transcriptional regulatory mechanism involved, is not known. Recent work on embryonal rhabdomyosarcomas has shown that the p38 MAPK signaling pathway mediated by the receptor for advanced glycation end-products could downregulate Pax7. Pax7 may reduce MyoD level by promoting its degradation in rhabdomyosarcoma [161]. Whether the similar degradation of MyoD controlled by Pax7 occurs in satellite cells remains to be examined. Pax7 has been shown to be able to activate the transcription of MyoD and Myf5 during embryonic development and in primary myoblasts [157, 160]. Whether Pax7-mediated MyoD degradation also occurs in satellite cells, in particular at the transition from a quiescent to an activated cell, requires further study. In addition, the relationship between Pax7’s role as a transcriptional activator of MyoD, and Myf5 expression and its function as a factor promoting MyoD degradation, are of interest and should be further explored.

Inhibitor of DNA binding (Id) proteins are negative regulators of MyoD [162]. Pax7 can also regulate the expression of Id proteins [163]. Sine oculis homeobox (Six) homeodomain transcription factors are also involved in satellite cell activation by regulating MyoD expression [164]. Six1 activates MyoD expression by directly binding the MyoD promoter [165]. MyoD expression is diminished by conditional depletion of Six1 in Pax7-expressing satellite cells [166].

## Satellite heterogeneity and satellite activation

Satellite cells are heterogeneous cell populations. They have various developmental origins, fiber associations, and expression profiles [10]. For example, the expression level of Pax7 varies dramatically in distinct subgroups of satellite cells. Pax7^High^ cells display reduced metabolic activity and delayed entrance into cell cycle upon stimulation, whereas Pax7^Low^ cells are more primed be activated [12]. A small proportion of quiescent satellite cells do not express CD34 and Myf5 [167], although the physiological significance of this has not been elucidated. The heterogeneity of satellite cells could affect the dynamics of satellite cell activation.

Myoblasts expressing desmin, MyoD, and myogenin have been observed within 12 h post-injury, when satellite cell activation is incomplete and proliferation has not yet been initiated [168]. The presence of the apparently differentiated myoblast population before satellite cell activation and proliferation could suggest the existence of two distinct satellite cell populations, the normal quiescent satellite cells and another group of more ready to differentiate [168]. Each population might be activated through different signaling pathways and display distinct kinetics. Identification of markers for each satellite cell subpopulation and development of methods for the specific isolation of each subpopulation will contribute to elucidate the specific activation mechanism governing each subpopulation. The accumulation of genomewide profiling at the single cell level will further deepen our understanding of the activation of heterogeneous satellite cells.

## Epigenetic regulation in satellite cell activation

### Histone modifications

Changes in histone modifications on Pax7, MyoD, Myf5, and other MRF genes occur during muscle regeneration. The Polycomb group (PcG) and Ttrithorax group (TrxG) epigenetic regulators play important roles in satellite cell activation and differentiation. PcG is composed of PRC1 and PRC2 complexes. KMT6, Ezh1, and Ezh2 are subunits of these two complexes with lysine methyltransferase activities. MLL1, MLL2, MLL3, MLL4, SET1a, and SET1b are subunits of TrxG complex with lysine methyltransferase activities. PcG marks chromatin with repressive H3K27me3 modification through its lysine methyltransferase activities whereas TrxG establishes the permissive H3K4me3 modification on chromatin [169, 170]. The transition from the transcriptionally permissive H3K4me3 modification to the repressive H3K27me3 modification is critical in cell fate determination [170]. This transition is induced by Ezh2 on the Pax7 gene upon satellite cell activation and during the subsequent proliferation stages, switching off Pax7 expression in a p38-MAPK-dependent manner [171]. The genes involved in cell cycle progression are also enriched for the permissive H3K4me3 modification in activated satellite cells [172].

Pax7 also participates in the establishment of the epigenetic pattern in satellite cell activation. In activated satellite cells and myoblasts derived from satellite cells, Pax7 recruits the Wdr5-Ash2L-MLL2 histone methyltransferase complex that methylates the H3K4 site to establish a permissive H3K4me3 modification on Myf5 [173].

A genomewide analysis of H3K4me3 and H3K27me3 profiles in purified quiescent satellite cells and activated satellite cells revealed a general lack of H3K27me3 repressive marker and the presence of H3K4me3 permissive marker on a large number of genes in quiescent satellite cells. Many non-myogenic-specific genes are labeled by the bivalent histone marker in quiescent satellite cells, which are replaced by the repressive H3K27me3 marker in activated satellite cells. The changes in the histone modification status in activated satellite cells compared with quiescent satellite cells may be due to the significant increase in the PcG subunit Ezh2 [123].

Sirt1, a member of the NAD^+^-dependent protein deacetylase family, is expressed in both quiescent and activated satellite cells. The reduction of Sirt1 level leads to premature differentiation [174], whereas its overexpression promotes satellite cell proliferation [175]. Sirt1 deacetylates MyoD and MEF2D to regulate their activities [176, 177]. Sirt1 interacts with Notch signaling pathway components Hes1 and Hey2 interfering with Notch activity [178]. This may also contribute to control of satellite cell activation (Fig. 1). Sirt1 can also serve as a nutrient sensor. Sirt1 interacts with ATG7 to activate autophagy through AMPK pathway. The activation of autophagic flux helps meet the high bioenergetic demands of satellite cell activation [179].

### DNA methylation in satellite cell activation

DNA methylation is an important aspect of epigenetic regulation. More than three decades ago, it was demonstrated that inhibition of DNA methylation transdifferentiated fibroblasts to muscle lineage [180]. In satellite cells, Dnmt-3b, a member of the DNA methyltransferase family, is recruited to CpG islands in Notch1 promoter in an Ezh2 binding-dependent manner to mediate increased methylation of Notch1 promoter under the control of TNF-α and NF-κB. Hyper-DNA methylation of Notch1 promoter mediated by TNF-α and NF-κB leads to a reduction of satellite cell self-renewal and proliferation [181]. The application of sulforaphane, a DNA methyltransferase inhibitor, to isolated satellite cells results in downregulation of the myostatin signaling pathway, which may promote satellite cell proliferation and differentiation [182].

The importance of DNA methylation in regulating stem cell functions has only begun to be unraveled in recent years and the data accumulated so far are limited. The current observations hint that DNA methylation might be an additional aspect to regulate satellite cell activation, but the direct link between satellite cell activation and DNA methylation is still missing. More systematic investigations are required to clarify the function of DNA methylation in this process.

### Non-coding RNA

Non-coding RNA (ncRNA) has been shown in the last decade to act as key regulators of gene expression. NcRNA makes up the majority of transcription products of the eukaryotic genome. It can be divided into structural and regulatory RNAs. Ribosomal RNA (rRNA), small nuclear RNA (snRNA), small nucleolar RNA (snoRNA), and tRNA are grouped as structural RNAs whose functions have been thoroughly studied for decades. Small regulatory RNA comprises microRNAs (miRNAs), piwi-interacting RNAs (piRNAs), and small interfering RNAs (siRNAs). Regulatory RNA molecules longer than 200 nucleotides are characterized as long non-coding RNA (lncRNA) [183]. In this review, we focus on the functions of miRNA and lncRNA in the process of satellite cell activation.

### Micro RNA

The myomiR family is a group of miRNAs specifically expressed in muscle, including miR-1a, miR-133, miR-206, miR-208, miR-486, and miR-499. The myomiR family is involved in regulating muscle differentiation and is capable of transdifferentiating fibroblasts to muscle lineage cells [184]. miR-1 expression is upregulated during muscle regeneration. It directly represses Pax7 expression to promote satellite cell proliferation and differentiation [185].

In addition to myomiRs, there are several other miRNAs involved in satellite cell activation. Both miR-31 and the mRNA of Myf5 are sequestered in ribonucleoprotein (RNP) granules, where miR-31 interacts with the 3’ UTR of Myf5 to suppress its translation in quiescent satellite cells [44, 186]. Upon satellite cell activation, Myf5 mRNA is released by downregulation of miR-31 and breakdown of RNP granules to rapidly switch on Myf5 protein translation [44, 186]. MiR-489 is enriched in quiescent satellite cells, and downregulated in activated satellite cells. It represses the expression of oncogene Dek to prevent quiescent satellite cells from entering cell cycle [187]. MiR-181 is upregulated during muscle regeneration. It targets Hox-A11, a negative regulator of MyoD, to promote satellite cell activation and differentiation [188]. MiR-206 is highly upregulated in activated satellite cells and myoblasts. It is also highly expressed in Duchenne muscles probably due to an intensified activation of satellite cells [186, 189–192]. Upregulation of miR-206 represses expression of Pax7, Notch3, IGFBP5, and Hmgb3 to promote satellite cell differentiation [193]. Downregulation of miR-125b occurs after injury to relieve IGF2 repression and promote subsequent satellite cell activation [194]. MiR-221/222 can also regulate satellite cell activation by promoting cell cycle progression [195, 196]. MiR-1192 can inhibit the translation of HMGB1, which is highly expressed in satellite cells and myoblasts, inducing the expression of myogenic factors such as MyoD and myogenin [197, 198].

A global downregulation of miRNAs has been observed in human satellite cells during the transition from quiescence to activation, in particular miR-106b, miR-25, miR-29c, and miR-320c [199]. The functions of these miRNAs remain to be elucidated.

### LncRNA

LncRNA can be grouped into cis-acting RNA and trans-acting RNA. Cis-acting RNA works in proximity to its transcription sites, whereas trans-acting RNA works at distinct loci. Both cis- and trans-acting lncRNA can recruit chromatin remodeling factors to alter the local or overall chromatin status to regulate transcription [200]. As the majority of the studies on lncRNA have been performed in C2C12 myoblast cell line, little is known about its functions in satellite cells.

The majority of known lncRNA molecules in myoblasts, such as Malat1, linc-MD1, SRA, Neat1, and YAM, are upregulated upon myoblast differentiation and are required for normal differentiation [201–207]. Linc-MD1 is located 13 kb upstream of pre-miR133b. Two microRNAs, miR133b and miR206, are located in the gene body of linc-MD1. Linc-MD1 fine-tunes the differentiation timing of myoblasts by sponging miR133b and miR206 to antagonize their repression on MAML1 and MEF2C [207]. The RNA-binding protein HuR binds linc-MD1 to facilitate the accumulation of linc-MD1 in cells and reinforce its sponge activity [201]. H19 is transcribed from the Igf2 locus and is highly expressed in adult muscle [208–210]. It interacts with PRC2 to repress the expression of Igf2, therefore inhibiting proliferation [211, 212]. H19 has several let-7 binding sites and serves as a microRNA let-7 sponge to relieve the inhibitory effects of let-7 on Igfbp2, an inhibitor of the IGF signaling pathway [213]. In myoblasts, H19 downregulates IGF signaling pathway to repress proliferation and may also play a similarly negative role in IGF signaling in satellite cells. To investigate the functions of lncRNA in satellite cell activation, expression profiles and functional assays should be carried out in quiescent and activated satellite cells.

The functions of ncRNA are just beginning to be realized. The specific functions of ncRNA during satellite cell activation, in particular, whether it can directly drive satellite cell activation, require further investigation.

## Conclusion

The activation of quiescent satellite cells is orchestrated by physiological cues, signaling pathways, and epigenetic regulators. We are just beginning to unravel how this process is regulated. Many questions remain unanswered, especially with regard to transcription and epigenetic regulation. Satellite cell activation is an asynchronized and transient process in vivo. Following the live cell activation process by high-resolution imaging and other new techniques will reveal more information about the process in vivo. The identification of more key genetic mutants affecting satellite cell activation will also help to reveal the missing links in the regulatory network, while genomewide analysis of the binding profile of epigenetic regulators will further deepen our understanding.

Many factors have been found to exhibit dramatic changes when satellite cells are activated. However, whether these changes are the causes of satellite cell activation or the consequences of it remains to be identified. Genetic mutations and careful characterization of the order of events during the satellite cell activation process will shed light on this. Another important question that remains to be explored is the link between uncontrolled satellite cell activation and cancer, especially non-alveolar rhabdomyosarcoma. Further exploration of this question will reveal more targets for drug development to treat rhabdomyosarcoma.
